# Mosaic theory revised: inflammation and salt play central roles in arterial hypertension

**DOI:** 10.1038/s41423-022-00851-8

**Published:** 2022-03-30

**Authors:** Felicitas E. Hengel, Jean-Pierre Benitah, Ulrich O. Wenzel

**Affiliations:** 1 III. Department of Medicine, University Hospital Hamburg-Eppendorf, Hamburg, Germany; 2 Inserm UMR-S 1180, Faculty of Pharmacy, University Paris Saclay, Gif-sur-Yvette, France

**Keywords:** Arterial hypertension, salt, innate and adaptive immunity, renin angiotensin aldosterone system, mineralocorticoid receptor, angiotensin II receptor, Inflammation, Adaptive immunity, Innate immunity

## Abstract

The *mosaic theory of hypertension* was advocated by Irvine Page ~80 years ago and suggested that hypertension resulted from the close interactions of different causes. Increasing evidence indicates that hypertension and hypertensive end-organ damage are not only mediated by the proposed mechanisms that result in hemodynamic injury. Inflammation plays an important role in the pathophysiology and contributes to the deleterious consequences of arterial hypertension. Sodium intake is indispensable for normal body function but can be detrimental when it exceeds dietary requirements. Recent data show that sodium levels also modulate the function of monocytes/macrophages, dendritic cells, and different T-cell subsets. Some of these effects are mediated by changes in the microbiome and metabolome due to high-salt intake. The purpose of this review is to propose a revised and extended version of the mosaic theory by summarizing and integrating recent advances in salt, immunity, and hypertension research. Salt and inflammation are placed in the middle of the mosaic because both factors influence each of the remaining pieces.

## Introduction

Arterial hypertension, which is commonly defined as blood pressure (BP) > 140/90 mmHg, affects more than a quarter of the global population, which is more than one billion people worldwide [1–3]. Due to multiple cardiovascular, renal, ocular and cognitive complications, arterial hypertension is a leading contributor to the global disease burden [1]. The limit of 140/90 mmHg was chosen according to multiple randomized controlled clinical trials that showed clear benefits of antihypertensive treatment relative to potential treatment-associated adverse effects in response to the primary preventative treatment of BP > 140/90 mmHg [3–5]. To account for the importance of BP elevations that are less than the usual classification of arterial hypertension, the American Heart Association and American College of Cardiology decided to reclassify arterial hypertension as BP > 130/80 mmHg in their latest guidelines in 2017 [6]. Under this definition, the prevalence of arterial hypertension in the United States has soared from 32 to 46% [6]. Despite the importance of arterial hypertension due to its high prevalence and immense impact on health worldwide, many details of the underlying pathological mechanisms leading to arterial hypertension remain unclear. This gap in knowledge is even more relevant when focusing on current antihypertensive treatment options, which mainly treat symptoms or have incompletely understood mechanisms of action. A better understanding of the pathogenesis of hypertension and the finely tuned interplay of key factors will lead to more precisely targeted therapeutic options with better global health outcomes and reduced side effects.

In the present review, we propose a revised, extended, and updated version of the mosaic theory that considers recent and exciting discoveries of the interactions among salt, inflammation, and hypertension. In this revised version of the mosaic theory, we place salt and inflammation in the middle of the mosaic because both factors influence each of the remaining tesserae.

## The multifactorial etiology of hypertension

**Fig. 1:**
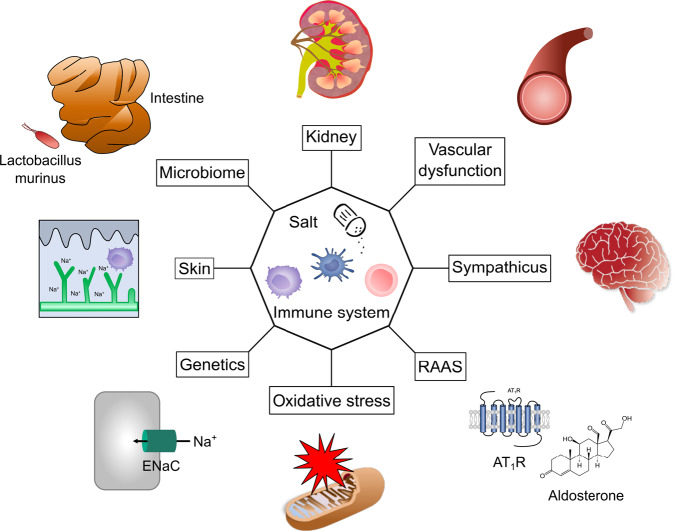
Revised version of the mosaic theory of hypertension. Salt and immune cells are placed in the center because they influence all other mosaic pieces. RAAS renin–angiotensin–aldosterone system, AT_1_R Ang II type 1 receptor, ENaC epithelial sodium channel

The multitude and interconnectedness of factors that influence BP were first recognized 80 years ago by Irvin Page, who postulated the impact of blood volume and viscosity, renal perfusion, vasculature, humoral factors and the central nervous system on BP. He realized the reciprocal influence of individual contributors on one another, composing a network of hypertension-inducing factors that is famously known as the *mosaic theory* of hypertension [7, 8]. Although several later revisions and many details have been added since its initial description, the principal idea of a complex system with interdependence among the individual components instead of simple cause-effect relationships of solitary sources of hypertension holds true today. An update of the *mosaic theory* was recently presented by Harrison et al. [9]. To underscore the influence of salt and inflammation on every piece of the *mosaic*, we would like to propose a further revised *mosaic theory* highlighting the central roles of salt and inflammation in the pathogenesis of arterial hypertension, as depicted in Fig. 1. The revised *mosaic theory* places salt and inflammation in its center; both factors influence all other mosaic pieces that have been proposed and are added by us. Notably, salt also influences innate and adaptive immune cells and vice ver*sa*. This extended *mosaic* will serve as a thread to discuss some of the individual key components. Another highly interesting and recent finding is the hypertensive response due to dehydration [10, 11]. However, further work needs to be done to add this interesting and fascinating theory to the mosaic.

The individual mosaic pieces are ordered clockwise according to the historical acknowledgment of their role in hypertension. The kidney was one of the first pieces of the mosaic to be recognized as crucial for BP regulation. Guyton developed the concept of pressure salt diuresis, establishing the link between BP, renal salt excretion and body fluid homeostasis [12]. Vascular dysfunction in arterial hypertension includes structural alterations in small and large arteries with increased vasoconstriction, endothelial dysfunction with impaired vasodilatation, and arterial wall thickening and stiffening by modifying the extracellular matrix and vascular smooth muscle cells [9, 13, 14]. We know that high-salt intake and inflammatory cells cause vascular stiffening [15, 16]. Sympathetic nerve (over)activity contributes to hypertension in several ways, including catecholamine-mediated vasoconstriction, vascular remodeling, adrenergic stimulation of renin production and secretion in juxtaglomerular cells, and increased sodium reabsorption and renal inflammation [17, 18]. The renin–angiotensin–aldosterone system (RAAS) is a crucial endocrine cascade that regulates water and electrolyte homeostasis, vascular tone, renal perfusion and cardiac remodeling, and its tight association with arterial hypertension was first noted in the 19th century and elaborated in the following decades [8, 19–22]. Recently, the influence the RAAS on immune cells was discovered [23]. The RAAS is discussed later in this review, with a particular focus on the role of the mineralocorticoid and Ang II type 1 (AT_1_) receptors in immune cells. The pleiotropic effects of vascular oxidation-induced reactive oxygen species (ROS) and oxidative stress on BP involving the vasculature, kidney, nervous system and immune activation have been extensively reviewed recently [24, 25]. Genetic polymorphisms in genome-wide association studies, familial hypertension and monogenetic disorders associated with a hypertensive phenotype underscore the importance of complex genetic influences on BP, which involve mutations in ion channels or receptor molecules that are important for renal sodium and potassium handling in the context of single gene mutations [9]. Almost all known monogenic blood pressure disorders of affect kidney salt metabolism. Open questions and the future (therapeutic) potential of hypertension genetics are elaborated elsewhere [26, 27]. Finally, the recently recognized role of monocytes/macrophages (M/Ms) in cutaneous salt storage and the response of the gut metabolome to salt yet another link between salt, systemic inflammation, and BP regulation and will be discussed further in this review.

## The immune system and hypertension

**Fig. 2:**
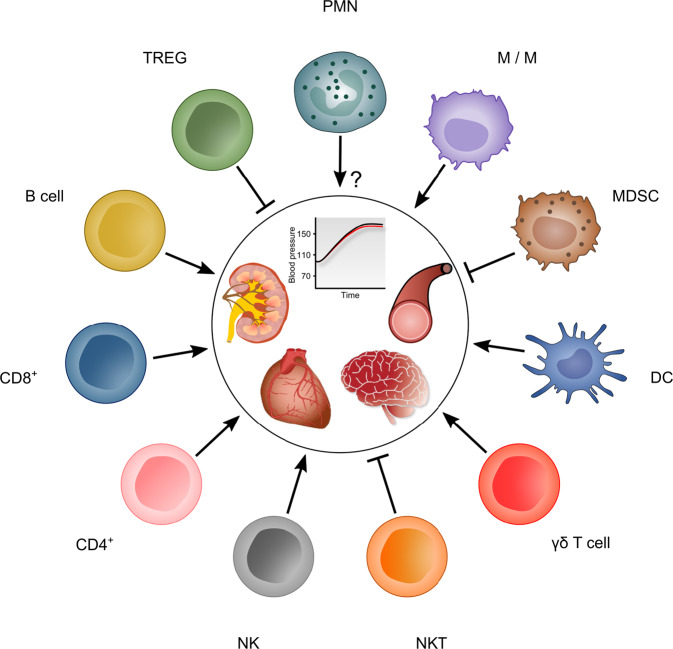
Overview of immune cells that influence BP- and hypertension-mediated end-organ damage. PMNs polymorphonuclear neutrophils, M/Ms monocytes/macrophages, MDSCs myeloid-derived suppressor cells, DCs dendritic cells, NKT cells natural killer T cells, NK cells natural killer cells, CD4^+^ cluster of differentiation 4-positive cells, CD8^+^ cluster of differentiation 8-positive cells, Tregs regulatory T cells

In recent years, increasing evidence of the immune pathogenesis of hypertension has emerged [28]. To review the complex connections between the immune system and hypertension, we will discuss the relevant individual components of the innate and adaptive immune systems, as visualized in Fig. 2.

### The innate immune system and hypertension

The innate immune system, which is characterized by rapid defense mechanisms against invading pathogens, consists of cellular and humoral components. The main cellular mediators are neutrophils, M/Ms, mast cells and innate lymphoid cells (ILCs, including killer-ILCs, which were formerly called natural killer cells and helper-like ILCs). Dendritic cells (DC), which are antigen presenting cells (APC), serve as a link between the innate and adaptive immune systems by processing foreign antigens and presenting the resulting antigenic peptides to T cells for their activation. Humoral factors of the innate arm of the immune system include the defensin and complement systems [29, 30].

#### The complement system

The complement system is a network of fluid-phase and membrane-bound proteins that detect pathogens, resulting in opsonization and the elimination of pathogens, as well as chemotaxis induced by anaphylatoxins to attract immune cells to the site of infection and induce a general inflammatory reaction. Cascade activation of these proteins triggers a highly amplified, instantaneous immune response that leads to the activation of C3- and C5-convertase complexes, which ultimately terminate in the formation of the membrane attack complex, which consists of the complement factors C5b to C9. Remarkable similarities in clinical and histopathological presentations of hypertension-induced malignant nephrosclerosis and atypical hemolytic uremic syndrome, which are complement-driven diseases, suggest a role of complement activation in the development and maintenance of hypertension. Elevated serum C3 and C5 levels in hypertensive patients and animal models of hypertension provide another hint of the role of the complement system in the etiology of hypertension. For further elaboration, the reader is referred to recent reviews with a particular focus on complement and hypertension [31–33].

#### Neutrophils

Polymorphonuclear neutrophils are part of the front-line defense of the innate immune system by rapidly eliminating microbes through phagocytosis, the release of antimicrobial proteins by degranulation and the generation of neutrophil extracellular traps. However, their role in the etiology of hypertension remains unclear. Epidemiological and animal data show an association between neutrophils, their activity and increased BP: epidemiological studies showed that the blood neutrophil-to-lymphocyte ratio, which is often used as a marker of systemic inflammation, correlates with the risk of developing hypertension and could serve as a predictor of hypertension [29, 34]. An animal model of spontaneously hypertensive rats exhibited significant associations between markers of neutrophil activity (myeloperoxidase activity and superoxide generation) and high BP [35]. However, these associations do not prove causality, and adoptive transfer of neutrophils did not restore the hypertensive response in myeloid cell-depleted mice [31]. Thus, neutrophils may not induce hypertension on their own, and their involvement with other cells that influence BP and factors that lead to hypertension remains to be further elucidated.

#### Monocytes/macrophages

Monocytes/macrophages (M/Ms), which are cells of the myeloid lineage, contribute to host defense through phagocytosis induced by pattern recognition receptors, antibodies or complement-mediated opsonization, as well as cytokine production and antigen presentation. Their role in the generation of hypertension was established using osteopetrotic mice that were deficient for macrophage colony-stimulating factor and subsequently had impaired M/Ms function. These mice exhibited reduced hypertension and vascular injury upon angiotensin II (Ang II) infusion or deoxycorticosterone acetate (DOCA) salt treatment [36, 37]. Wenzel et al. found attenuated infiltration of the vascular wall after Ang II treatment in mice with selective ablation of lysozyme M-positive myelomonocytic cells, accompanied by reductions in oxidative stress, vascular dysfunction and hypertension [38]. This attenuation was reversible upon the adoptive transfer of monocytes. Renal infiltration and the proliferation of macrophages is associated with interleukin (IL)-6 expression and can be reduced by anti-IL-6 treatment [39]. Furthermore, monocytes, particularly proinflammatory M1-like macrophages, express angiotensin-converting enzyme (ACE), indicating another direct way these cells can influence BP by activating the RAAS [40, 41]. For further elaboration on the role of macrophage polarization in hypertension, please refer to Harwani et al. and Wenzel [42, 43].

IL-6 is a pivotal cytokine associated with innate immunity that plays a proatherogenic role in cardiovascular disease, and IL-6 inhibition may be a novel strategy to exert cardiovascular protection. IL-6 signaling and anti-IL-6 treatment have recently been reviewed by Ridker and Rane [44]. Ziltivekimab, a novel IL-6 ligand antibody, has been developed specifically for use in atherosclerotic diseases and is poised to be tested in a large-scale cardiovascular outcome trial focused on individuals with high atherothrombotic and inflammatory risk due to chronic kidney disease and elevated levels of CRP [44, 45].

#### Myeloid-derived suppressor cells

Myeloid-derived suppressor cells (MDSCs) are a heterogeneous population of myeloid progenitor cells and immature myeloid cells with immunosuppressive capacities [29, 46, 47]. These cells are characterized by the expression of the myeloid markers CD11b and Gr1 (in mice) and can suppress T-cell activation and TH17 and T_H_1 cells [47, 48]. Established mechanisms by which MDSCs suppress T-cell activity include the enzymes arginase 1 (which depletes the nonessential amino acid l-arginine, whose depletion in turn inhibits T-cell proliferation) and inducible nitric oxide synthase (which generates NO, which inhibits T-cell function via the Janus kinase JAK3 and signal transducer and activator of transcription (STAT5), as well as MHC II expression and apoptosis induction), as well as powerful oxidants such as ROS and peroxynitrite [47]. Furthermore, these cells can induce the differentiation of regulatory T cells (Tregs) by producing interleukin 10 (IL-10) [48]. Through their anti-inflammatory effects, MDSCs play a protective role and counteract the development of hypertension: different mouse models of hypertension showed increased numbers of CD11b^+^Gr1^+^ myeloid cells, and the depletion of these MDSCs enhanced renal inflammation and BP. Adoptive transfer of MDSCs from hypertensive but not normotensive mice reduced Ang II-induced hypertension [49]. These antihypertensive effects were mediated at least partially by ROS generation, and MDSCs are an interesting new addition to the story of how the immune system and ROS influence and classically increase BP.

#### Dendritic cells

**Fig. 3:**
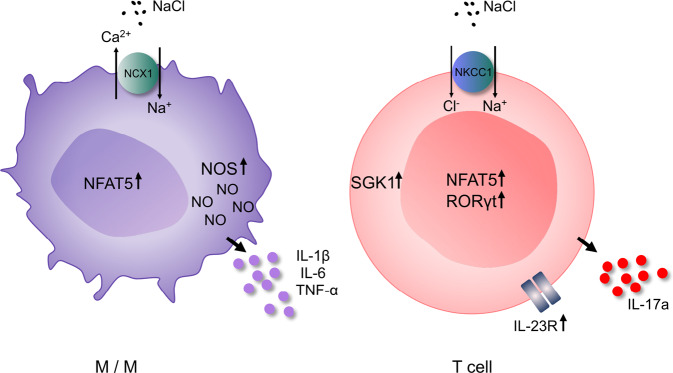
In monocytes/macrophages, high-salt enters proinflammatory cells via NCX1, causing NFAT5 expression and subsequent NO production by NOS2. In addition, proinflammatory cytokines such as IL-1β, IL-6, and TNF-α are released in response to high salt. In T cells, sodium enters the cell through the NKCC1 transporter, resulting in the upregulation of NFAT5 and its downstream target SGK1. Upregulation of the T_H_17 transcription marker RORγt mediates the transcription and translation of IL-23R, which is essential for T_H_17 induction and the secretion of IL-17a. NCX1 sodium calcium exchanger 1, NKCC1 sodium potassium chloride cotransporter, NOS2 nitric oxide synthase, NO nitric oxide, IL interleukin, TNF tumor necrosis factor, SGK1 serum glucocorticoid-regulated kinase, NFAT5 nuclear factor of activated T cells, RORγt retinoic-acid receptor-related orphan nuclear receptor gamma, M/M monocyte/macrophage

DCs contribute to the inflammatory response associated with hypertension via T-cell activation. This effect is induced by antigen presentation and costimulation of T cells by B7 ligands (cluster of differentiation (CD)-80 and CD86) interacting with the T-cell costimulation receptor CD28 (Fig. 3). Mice with experimentally induced hypertension due to Ang II infusion or DOCA salt treatment exhibit increased numbers of DCs in secondary lymphatic tissues. DCs secrete cytokines such as IL-1, IL-6, and IL-23 and can activate CD8^+^ and CD4^+^ T cells in renal lymph nodes or other tissues, and these activated T cells can then migrate into the kidney and vasculature. Inhibition of B7-induced costimulation by CTLA4-Ig (abatacept) reduces vascular T-cell infiltration and activation, leading to marked alleviation of hypertension in these mouse model [50]. In knockout mice lacking B7, Ang II infusion was incapable of inducing hypertension [50]. To rule out the influence of B7 on endothelial cells, these knockout mice underwent wild-type bone marrow transplantation, which reconstituted Ang II-induced increase in BP [50]. Hevia et al. confirmed these findings by showing that specific ablation of CD11c-expressing APCs, which characterizes DCs, prevented the development of hypertension after Ang II infusion on a high-salt diet and improved natriuresis [51]. The researchers found a decrease in anti-inflammatory effects, as measured by decreased cardiac and renal transcription of IL-10 and the master transcription factor of regulatory T cells, forkhead box P3 (FoxP3), and this effect was prevented by DC ablation. Furthermore, the researchers showed an increase in CD86 in splenic and renal DCs due to Ang II and salt treatment. In vitro, these DCs induced a stronger proinflammatory response characterized by increased T-cell proliferation and differentiation toward CD8^+^/IFN-γ cells than those from control mice without Ang II infusion and with a regular salt diet [51]. However, given the expression of CD11c in other cell types, such as macrophages, further studies with specific DC-knockout could improve our understanding of the influence of different APC or DC subtypes on BP [52]. Lu et al. additionally showed that mice lacking renal DCs had increased sodium chloride cotransporter expression and enhanced natriuresis and diuresis, as well as reduced urinary oxidative stress markers compared to wild-type mice with Ang II and salt treatment [53].

However, the influence of DCs and hypertension is bidirectional, resulting in a self-enhancing interplay: DCs not only exacerbate hypertension, but hypertension enhances DC activity and activation. Hypertensive mechanical trauma might produce neoantigens due to cell death and the subsequent exposure of intracellular antigens, which are then presented to T cells by DCs. Furthermore, increased oxidative stress due to arterial hypertension results in lipid oxidation with the formation of isoketal adducts of various self-proteins. Highly reactive isoketals can ligate to the lysine residues of proteins in DCs, forming protein adducts that are recognized as nonself. These proteins then act as damage-associated molecular patterns and stimulate the production of cytokines, including IL-1β, IL-6, and IL-23; these proteins are then processed into peptides and presented to T cells. The resulting T-cell activation stimulates IFN-γ and IL-17A production and exacerbates hypertension, which was shown by transferring isoketal-activated DCs into wild-type mice, leading to a rise in BP [54]. The control group of Rag1^−/−^ mice lacking T cells did not develop hypertension. Consistently, treatment with isoketal scavengers prevented hypertension and associated renal damage in animal studies [54]. Thus, DCs mediate the development of hypertension through proinflammatory T-cell activation and enhance oxidative stress, as well as fluid and sodium retention.

#### γδ T cells

The unconventional and rather small subgroup of γδ T cells has receptors composed of γ- and δ-chains and plays a role in hypertension by producing IL-17A and IFN-γ (for further elaboration of the hypertensive effects of IL-17A and IFN-y, see CD4^+^ T cells). Caillon et al. showed that Ang II-induced hypertension was paralleled by an increase in the number and activation of γδ T cells and that knockout or antibody-mediated γδ T deficiency prevented Ang II-induced hypertension, endothelial dysfunction and splenic T-cell activation in mice [55]. In humans, there was an association between blood γδ T-cell frequency and (rising) BP. γδT cells are also involved in the inflammatory infiltration of the hypertensive heart, and γδT-cell depletion prevented Ang II-induced cardiac injury in mice [56].

#### Natural killer T cells

Although further studies are necessary, the first evidence from CD1d-knockout mice suggested a protective role of natural killer T cells (NKTCs) in the development of hypertension. Cd1d knockout in APCs leads to impaired NKTC activation and subsequent reductions in NKTC activity. Wang et al. showed increases in Ang II-induced hypertension, cardiac inflammation, hypertrophy and fibrosis in mice with impaired NKTC activity and IL-10 production [57]. Adoptive transfer of CD1d-knockout bone marrow to wild-type mice confirmed these findings. Activating NKTCs specifically with α-galactosylceramide or recombinant murine IL-10 improved Ang II-induced hypertension, cardiac function and unfavorable remodeling.

#### Natural killer and innate lymphoid cells

Natural killer (NK) cells have cytotoxic functions and a large array of activating and inhibitory receptors are distinct from ILCs, which are the most recently discovered family of innate immune cells. The latter are primarily tissue-resident cells with innate and adaptive features that respond to pathogenic tissue damage by secreting signaling molecules and cytokines. These properties may predispose ILCs to play roles in the development of hypertension, which has not been studied thus far. In comparison, a proinflammatory effect of classic NK cells in hypertensive cells has already been suggested; Ang II-induced the proliferation and migration of NK cells in vitro and their recruitment to the aortic wall, causing endothelial dysfunction in vivo, and this effect was blunted by the presence of anti-NK cell antibodies [29, 58]. Additional studies are needed to further characterize the role of NK cells in hypertension and to elucidate the effects of other ILC types on BP and the cardiovascular system.

### The adaptive immune system and hypertension

The first conclusive evidence for the role of the adaptive immune system in the pathogenesis of arterial hypertension was provided by Guzik et al. and showed that the increase in BP caused by Ang II infusion was significantly blunted in mice lacking T and B cells [59]. This finding was confirmed in several other laboratories and genetic models [60–63]. Surprisingly, we did not observe resistance to the effects of Ang II in B6.Rag1^−/−^ mice, which do not have mature T and B cells [64]. The Sandberg laboratory also reported that Jackson B6.Rag1^−/−^ mice lost their resistance to Ang II-induced hypertension later on [65]. While it is clear that lymphocytes play a critical role in hypertension, the negative data illustrate that there are important but unidentified confounders. Possible confounders range from the loss of phenotype due to compensatory mechanisms in the immune system over generations of laboratory breeding to undiscovered environmental factors, which might change over time and between different laboratories, such as regular chow and/or alterations in the microbiota [63, 66, 67]. Under the hypothesis of compensatory mechanisms, one could even consider on the possibly of even greater importance of the immune system in BP regulation since it seems to be able to regain its effect on BP after initial knockout. Taken together, this ambiguity sparks even more interest in disentangling the complex relations among immune cells and arterial hypertension, which is why we will discuss the latest insights into how cells of the adaptive immune system regulate BP.

#### CD4^+^ T cells

Depending on antigen stimulation and costimulation, as well as exposure to specific cytokines, naïve CD4^+^ T-helper cells differentiate into different lineages, as determined by master transcription factors that are activated by signaling transducer and activator of transcription (STAT) signaling. The resulting subpopulations are T_H_1, T_H_2, and T_H_17 cells, Treg cells and follicular helper T cells, which have specific cytokine profiles and functions [68]. As early as 1976, Svendsen demonstrated the need for T cells ni the development of chronic hypertension and renal end-organ damage in response to DOCA salt treatment in athymic mice, and DOCA salt treatment induced a prolonged increase in BP only after thymus transplantation from wild-type mice [69]. This finding of the need for T cells in hypertension was confirmed by Guzik et al. in T- and B-cell-deficient mice, which developed Ang II-induced hypertension, endothelial dysfunction, vascular remodeling and increased oxidative stress only upon adoptive transfer of T cells [59]. Salt-sensitive rats showed a significant increase in renal T-cell infiltration and oxidative stress with increasing salt intake, which was blunted by immunosuppressive treatment with the calcineurin inhibitor (CNI) tacrolimus for 3 weeks [70]. Tacrolimus treatment also significantly reduced high-salt-induced mean arterial blood pressure and albumin excretion in this study, which serves as proof of concept that calcineurin inhibition antagonizes salt-induced hypertension. The clinical effects, however, are not as straightforward: data from several studies showed reductions, no changes or increases in blood pressure in response to CNI treatment [71]. These clinical effects were probably due to the long-term off-target effects of CNIs, which enhance hypertension by increasing sympathetic activity, endothelin production, renal afferent vasoconstriction, and salt retention [71]. In addition to oxidative stress, T cells seem to influence BP via cytokine-dependent regulation of renal sodium reabsorption [72]. Mice that are deficient in IFN-γ, the major cytokine of T_H_1 cells, showed differences in the abundance and phosphorylation of renal ion transporters after Ang II infusion compared to wild-type mice. In addition, these mice showed decreased levels of sodium-proton-exchanger (NHE) in the proximal tubule, sodium potassium chloride cotransporter (NKCC) in the thick ascending limb and sodium chloride cotransporter (NCC) in the distal tubulus, resulting in a blunted hypertensive responses [72]. NHE and the overall hypertensive response to Ang II were also reduced in IL-17-deficient mice [72]. Another study confirmed that IL-17A deficiency abolished the activation of NCC and the epithelial sodium channel (ENaC) and blunted glomerular and tubular injury in Ang II-infused mice. In vitro treatment of distal convoluted tubule cells with IL-17A increased NCC activity via the phosphorylation of serum glucocorticoid-regulated kinase 1 (SGK1), a downstream target of osmoregulatory and proinflammatory signaling pathways (also see Salt, immune cell activation, and antimicrobial defense). These effects were countered by SGK1 inhibition [73]. IL-17A, which is mainly produced by proinflammatory T_H_17 cells, is a key mediator of renal and vascular dysfunction in hypertensive mice, correlates with hypertension in humans and constitutes a promising target for new therapeutic approaches, such as IL-17 antibodies, as these agents are already approved for certain autoimmune diseases [74].

#### CD8^+^ T cells

Further evidence for a role of T-cell-driven inflammation in human hypertension stems from Youn et al. who observed a T-cell population with an increased fraction of immunosenescent, proinflammatory cytotoxic CD8^+^ T cells in the peripheral blood samples of patients with newly diagnosed hypertension [75]. CD8^+^ T cells constitute a major source of IFN-γ, and hypertensive patients showed increased numbers of CD8^+^ T cells expressing IFN-γ, granzyme B, perforin, and tumor necrosis factor alpha (TNF-α). Granzyme B, a serine protease, and perforin, a pore-forming protein, are involved in cell-mediated cytotoxicity. Granzyme B is further thought to be involved in matrix remodeling, fibrosis, and the disruption of endothelial barrier function. Serum granzyme B levels were elevated in hypertensive patients, and Shen et al. found that granzyme B deficiency protected against Ang II-induced cardiac end-organ damage, including hypertrophy, fibrosis, and inflammation [76, 77]. CD8^+^ deficiency in mice also prevented Ang II-induced endothelial dysfunction and renal vascular remodeling and rarefaction, leading to the alleviation of Ang II-induced hypertension [78]. CD8^+^-deficient mice showed superior renal sodium excretion upon artificial sodium loading, suggesting a role for cytotoxic CD8^+^ T cells in sodium retention. The previously mentioned effect of CD8^+^ T-cell-derived IFN-γ on renal sodium transporters, particularly NHE in the proximal tubule, NKCC in the thick ascending limb and NCC in the distal tubulus, might be one reason for this observation [72]. Another way CD8^+^ T cells can influence renal sodium resorption was observed by Liu et al.: direct contact of CD8^+^ T cells with cells of the distal convoluted tube stimulated the expression of NCC and the development of salt-sensitive hypertension in DOCA salt-treated mice [79]. In vitro, this upregulation was shown to involve ROS-induced activation of tyrosine-protein kinase Src (also called cSrc, cellular and sarcoma) and the subsequent upregulation and stimulation of potassium and chloride channels to facilitate increased sodium reabsorption. Spatial separation of CD8^+^ T cells and tubular epithelial cells abolished the observed effect, strongly supporting the necessity of direct physical contact [79].

#### B cells

Although the adoptive transfer of B cells alone was not able to ameliorate hypertension in T-cell- and B cell-deficient mice, accumulating evidence supports the role of B cells in the etiology of hypertension. Chen et al. demonstrated an increase in IgG and the accumulation of activated B cells, plasmablasts and plasma cells in the aortic wall upon Ang II infusion [80]. Mice with B-cell deficiency due to knockout of the B-cell activating factor receptor (Tnfrsf13c^−/−^ mice) or the transcription factor Myb (c-myb^h/h^ mice), as well as mice treated with anti-CD20 antibody therapy, were protected against Ang II-induced hypertension and showed reduced aortic macrophage infiltration, collagen deposition and vascular stiffening [80, 81]. In vitro treatment of macrophages with purified IgG from hypertensive mice induced the production of the profibrotic transforming growth factor (TGF)-β. The protective effect was reversed by adoptive transfer of wild-type B cells into Tnfrsf13c^−/−^ mice [80]. Depletion of plasma cells by the proteasome inhibitor bortezomib showed similar beneficial results [82]. These results are consistent with long-standing findings that patients with essential and pregnancy-related hypertension have elevated circulating levels of IgG and IgM in comparison to normotensive individuals, and hypertensive patients are prone to develop circulating autoantibodies against a variety of autoantigens, including AT_1_ receptors, adrenoceptors and voltage-gated calcium channels [83–86]. Since B cell-targeted therapies are frequently used in clinical settings, the effect of anti-CD20 therapy on blood pressure in humans is of high interest. Data on the effects on blood pressure, however, are often confounded by polypharmacy. Studies without polypharmacy consistently showed a short-term reduction in BP in response to anti-CD20 treatment, but long-term results were inconsistent [71].

#### Regulatory T cells

Regulatory T cells (Tregs) are essential immunosuppressive regulators of the immune response and inflammation that induce and maintain immunological tolerance mainly through IL-10 production. Recent data suggest a protective role of Tregs in the etiology of hypertension, particularly by regulating renal and vascular inflammatory processes. The first evidence was provided by animal studies that showed an association between hypertension and decreased Treg counts in peripheral blood and the kidney [76, 87–92]. Although these findings were not reproducible in all mouse models of hypertension and in humans, the protective effects of Tregs against hypertension is underscored by several other findings [76, 93]. Vascular damage induced by Ang II infusion was exacerbated in Rag1^−/−^ mice with T-cell deficiency and T-cell reconstitution from mice lacking Tregs due to a mutation in the master transcription factor of Tregs, FoxP3 [94]. Consistently, IL-10-deficient mice exhibited exacerbated endothelial dysfunction and increased superoxide production in response to Ang II [95]. Studies on the adoptive transfer of Tregs in Ang II- or aldosterone- and salt-treated mice yielded heterogeneous results regarding BP reductions, but a robust decrease in hypertension-induced end-organ damage, such as vascular stiffening and cardiac hypertrophy was observed [76]. Chen et al. further examined the relationship between Ang II-induced damage and Treg function and suggested a role of complement, particularly the binding of the anaphylatoxins C3a and C5a to their receptors on Tregs, in Ang II-mediated impairment of Treg function. The researchers found decreased expression of FoxP3 in response to Ang II treatment [88]. Knockout of C3a and C5a receptors alleviated Ang II-induced hypertension and renal and vascular damage. Adoptive transfer of C3a- and C5a-deficient Tregs protected against Ang II-induced hypertension [88]. Another Treg-dependent way to protect against hypertension and subsequent damage was demonstrated by Taylor et al. [96] and Majeed et al. [97] who showed that selective Treg expansion by infusion of low-dose IL-2 attenuated BP increases and renal damage in a mouse model of SLE, inhibited aortic collagen remodeling and resulted in vascular stiffening in Ang II-treated mice [96, 97]. Furthermore, cotreatment with IL-10 and IL-4 prevented preeclampsia in pregnant mice [98]. Taken together, these data strongly support a beneficial, anti-inflammatory role of Tregs in the etiology of hypertension and particularly in subsequent end-organ damage. Further elucidation of the underlying pathological mechanisms might provide new therapeutic approaches, including the expansion, stimulation or even application of Tregs and associated cytokines, which is a promising outlook, especially in light of currently expanding Treg-based therapies in the treatment of autoimmune diseases, transplant rejection and graft-versus-host disease [99].

The influence of the immune system on cardiovascular health involves not only the development of hypertension but also the chronic inflammation driving atherosclerosis and cardiovascular disease (CVD) [100]. Research on systemic anti-inflammatory treatment of CVD first targeted C-reactive protein in rats with acute myocardial infarction (MI) to limit infarct size and cardiac dysfunction (39). Further research now shows promising results in reducing cardiovascular morbidity and mortality. The monoclonal antibody canakinumab, which targets IL-1β, could reduce recurrent cardiovascular events in a high-risk population with previous MI and elevated CRP [101]. Similarly, continued colchicine treatment after MI decreased the risk of ischemic cardiovascular events [102]. However, this systemic anti-inflammatory treatment comes at the cost of increased susceptibility to (fatal) infectious complications [101]. To fully exploit the beneficial effects of immunosuppression on cardiovascular health while avoiding (infectious) side effects, a better understanding of the underlying pathological mechanisms and, ultimately, more specific anti-inflammatory treatment options are needed.

## Salt

Salt plays a central role in the development and maintenance of hypertension, as highlighted in our revised version of the mosaic theory shown in Fig. 1. Consumption of the modern diet has led to a worldwide increase in sodium intake to levels above the biologically required and recommended limits. These eating habits can promote hypertension and cardiovascular disease [103, 104]. However, there is still a vigorous debate about optimal salt intake and the importance of dietary salt reductions, which were recently fueled by a global observational study that showed a positive correlation between salt intake and life expectancy and an inverse correlation with all-cause mortality [105]. Such an emotional discussion is rather unusual in scientific questions. Could the emotional energy surrounding the issue of salt be due to the cultural and historical significance of this substance over the last several thousand years [106, 107]?

Salt has a long history with mankind. The harvest of salt from the surface of Xiechi Lake near Yuncheng in Shanxi, China, is one of the oldest verifiable saltworks and dates back to 6000 B.C. [108]. The Latin terms for health and healthy, “salutem” and “salubris”, are derived from the word “sal” (salt). Approximately 2700 years B.C. The PENG-TZAO-KAN-MU was published in China. It is the earliest known treatise on pharmacology, and a large part was devoted to the discussion of more than 40 kinds of salt. This discussion included descriptions of salt extraction and manufacturing methods that show amazing similarity to processes used today [108]. Salt products were rare and expensive and brought wealth and power to its owners. It is thought that much of the construction of The Great Wall of China was paid by the salt tax [108]. Salt was used as money in many regions of the world, and salt money has recently been found in Africa and China [108].

### Human evolution and salt intake

Hominid evolution began 30 million years ago in warm regions and was associated with a vegetarian diet and low salt intake. Adequate salt intake is important in tropical areas to enable acclimatization to intense sweating [109]. The development of salt saving mechanisms would thus have been favored by selection pressure, as an adequate amount of salt is indispensable for life. The consequences of salt consumption were of peculiar interest long ago. The famous Yellow Emperor wrote in ~3000 B.C.: “If too much salt is used in food, the pulse hardens, tears make their appearance and the complexion changes” [110]. However, only recently in the 19th century did the idea that too much salt intake could be harmful arise [109]. Since then, this harm has been investigated further. Excess salt ingestion can have profound health consequences, including hypertension and cardiovascular target organ damage. Several potential pathophysiological mechanisms relating a high-salt diet to cardiovascular disease have been characterized and include volume expansion and changes in the RAAS and ROS. Salt increases BP and induces damage in the target organ at least in part by polarizing adaptive and innate immune cells toward a proinflammatory phenotype, which is discussed in detail below.

### New salt-associated concepts: Salt, inflammatory cells, and hypertension

Salt, which is a central component of the *mosaic theory* of hypertension, was long known to be an important factor in the etiology of hypertension; early concepts of renal hypertension suggested that insufficient renal sodium excretion led to the expansion of extracellular fluid volume during high-salt intake and a subsequent rise in BP due to increased cardiac output and an adaptive increase in peripheral resistance [9, 12]. Guyton noted the increase in salt excretion with increased BP [12]. Additionally, salt sensitivity in arterial hypertension was discovered early in salt feeding experiments with rats and was later shown to affect ~25% of normotensive and 50% of hypertensive humans, with an increased prevalence in the elderly population [9, 111, 112]. Salt sensitivity is characterized by the enhanced dependence of BP on dietary salt intake of affected individuals, and an increase in dietary salt results in a parallel rise in BP [112]. Overreactivity of the RAAS and sympathetic nervous system, vascular dysfunction with increased peripheral resistance and endothelial stiffness in response to high sodium, insufficient negative tubuloglomerular feedback of the macula densa and atrial natriuretic peptide are among the reasons for impaired renal sodium excretion in salt-sensitive hypertension [112]. For further details, the reader is referred to the recent review by Ellison and Welling on salt handling and blood pressure [113]. Accordingly, epidemiological genomic studies showed a moderate heritability of salt sensitivity and the associations of different genetic variants affecting the RAAS, ion channels involved in sodium and water regulation, endothelial components, and gene products involved in the generation of NO and ROS [114]. The multitude of associated genetic variants hints at the complexity with which salt influences BP. Over the last decades, our understanding of the complex influence of sodium on BP has been broadened, and many new and fascinating details have been illuminated further.

An important finding in recent years is that salt can directly modulate the phenotypes of myeloid cells and different subsets of T cells, as reviewed recently by Rucker et al. and Wilck et al. [103, 115]. Whether salt-induced immunomodulation leads to beneficial or harmful effects on the body might depend on the specific immunological context and is explained in more detail below.

#### Cutaneous salt storage

One new approach to explain salt sensitivity and its increase with age is an impaired or exhausted ability to balance total body sodium over time. Sodium is the most abundant cation in extracellular fluid, which consists of the interstitial and intravascular fluids. Contrary to the initial assumption of iso-osmolarity in those two fluid compartments, the idea of hypertonic sodium storage in interstitial fluid in the skin, which is controlled and drained into the intravascular fluid by lymphatic vessels, emerged, since rodents fed high sodium exhibited hypertonic interstitial volume retention [116]. This finding triggered some interest to improve understanding of the effect of altered ion availability on the biology of immune cells in maintaining inflammatory, infectious, and homeostatic processes [117–119]. High sodium concentrations in turn are recognized as chemotactic stimuli by macrophages in a dose-dependent manner, as depicted in Fig. 3 [120]. Macrophages can sense high sodium concentrations via sodium calcium exchanger 1 (NCX1), resulting in macrophage activation and the subsequent activation of the osmoprotective transcription factor nuclear factor of activated T cells 5 (NFAT5, also called TonEBP) [121]. NFAT5 leads to increased production of NO by nitric oxide synthase (NOS)-2 and the production of proinflammatory cytokines such as IL-1β, IL-6 and TNF-α, which are released in response to high salt [122]. Furthermore, NFAT5 triggers the secretion of vascular endothelial growth factor (VEGF)-C [116, 121]. VEGF-C signaling in turn results in VEGF receptor (VEGFR) 3-dependent hyperplasia of the lymph capillary network, which enhances interstitial sodium clearance by improving the drainage of interstitial fluid and electrolytes into the intravascular compartment. VEGF-C also stimulates the expression of endothelial nitric oxide synthase (eNOS) via the VEGFR2 receptor, increasing NO production and acting as a direct compensatory vasodilatory mechanism to buffer the increase in BP due to excess extracellular volume [116]. Furthermore, a disturbance in macrophage infiltration or VEGF-C signaling increases BP in rats fed a high-salt diet [116].

In addition to the central role of macrophage and dendritic cell signaling in osmotic homeostasis, the importance of glycosaminoglycans (GAGs) in the regulation of interstitial sodium storage was postulated by Titze et al. [123]. A hypertonic, osmotically inactive (e.g., water-free) sodium concentration of up to 190 mmol/l was found to be associated with skin levels of GAG, which might be responsible for binding sodium via its negative charge [123]. Changing the skin levels of GAG by inhibiting or stimulating GAG chain polymerization might be a means of regulating skin as a potential sodium reservoir [123]. Accordingly, ^23^Na-MRI visualization of skin sodium levels showed increased sodium accumulation with age, as well as in patients with refractory hypertension, and skin biopsies of healthy volunteers revealed an increase in skin sodium concentrations that paralleled the increase in dietary salt intake [124, 125].

In T cells, sodium enters the cell by the NKCC1 transporter, which can be inhibited by furosemide. Sodium upregulates the expression of NFAT5 and its downstream target SGK1. SGK1 phosphorylation subsequently enables retinoic-acid receptor-related orphan receptor gamma t (RORγt)-mediated transcription of the IL-23 receptor, which is essential for T_H_17 polarization (Fig. 3) [103].

#### Salt, immune cell activation, and antimicrobial defense

In addition to its role in BP regulation, locally increased sodium concentrations in the skin were thought to serve another purpose; Jantsch et al. found local sodium accumulation in skin infection sites in humans and mice, strengthening antimicrobial host defense by activating macrophages via NFAT5, enhancing NO production and targeting pathogens to autolysosomes [122, 126]. Ultimately, a high-salt diet improved antimicrobial control and healing in mice infected with *Leishmania major* [122]. Accordingly, sodium chloride treatment of macrophages followed by activation via lipopolysaccharide (LPS) or CD4^+^ T-cell interactions in vitro stimulated a proinflammatory phenotype with enhanced production of the proinflammatory cytokines TNF-α and interleukin 12 (IL-12), promoted the differentiation of “M1-like” macrophages and reduced M2-like regulatory macrophage activity to suppress T-cell proliferation [127, 128]. The downside of this salt-induced macrophage boost lies in macrophage-driven autoimmunity. Mice with experimental autoimmune encephalomyelitis (EAE, a mouse model of multiple sclerosis) on a high-salt diet exhibited increased macrophage infiltration and activation within the central nervous system, and transferring macrophages that were treated ex vivo with excess amounts of sodium chloride exacerbated the disease state [127]. In addition, glucocorticoids are increased during a high-salt diet in mice and humans. This effect is a direct consequence of the downregulation of aldosterone synthase, which causes the accumulation of corticosterone, which is an aldosterone precursor with glucocorticoid functionality [129].

Consistent with macrophage infiltration and activation, Kleinewietfeld et al. and Wu et al. showed increased induction and infiltration of T_H_17 cells in the central nervous system in mice with EAE that were fed a high-salt diet [130, 131]. Exposure to excess sodium chloride in vitro enhanced the polarization of naïve CD4^+^ cells to T_H_17 cells and upregulated the production of the proinflammatory cytokines IL-17A, granulocyte-macrophage colony-stimulating factor (GM-CSF), TNF-α and IL-2 [131]. This induction of T_H_17 cells is mediated by the osmoprotective transcription factor NFAT5 and its downstream target SGK1, which deactivates the transcription factor forkhead box protein O1 (FOXO1). Together with the upregulation of the T_H_17 transcription marker RORγt, this leads to enhanced transcription and translation of the IL-23 receptor and subsequent T_H_17 cell activation by IL-23 [130]. Biochemical or genetic ablation of NFAT5 or SGK1 abolishes salt-induced T_H_17 differentiation [131]. Furthermore, high sodium chloride conditions compromise regulatory T-cell function by reducing IFN-γ production via SGK1 [132]. SGK1 is also involved in osmoregulation as a target of mineralocorticoid receptor (MR) signaling. Indeed, SGK1 is responsible for the mineralocorticoid-induced increase in renal epithelial sodium channel ENaC activity by slowing its degradation. Therefore, SGK1 is essential for antinatriuresis under low salt conditions, and urinary sodium loss and decreases in BP and the glomerular filtration rate have lethal consequences in SGK1-knockout mice under salt restriction [133].

A high sodium concentration can further promote T-cell stimulation and increased IFN-γ and IL-17A production by boosting DC activation: DCs sense environmental sodium via amiloride-sensitive sodium channels, specifically NHE1 and ENaC [134]. Sodium influx elicits calcium influx by plasma membrane-bound NCX1, and calcium in turn activates protein kinase C (PKC) to phosphorylate NADPH oxidase (NOX) 2 subunits. The subsequent increased production of superoxide promotes the processing and presentation of self-antigens by DCs, resulting in autoimmune-like inflammation and dysfunction in the kidney and vasculature. Transferring high sodium-treated DCs into naïve mice and infusing subpressor doses of Ang II resulted in hypertension, and this effect was not seen after the transfer of DCs exposed to normal sodium concentrations [134].

An effect of sodium on the complement system was discovered when renin-mediated cleavage of C3 into C3b and C3a linked sodium-dependent RAAS activity to complement activation [135]. The detailed role of the complement system in arterial hypertension itself remains unclear and is comprehensively reviewed elsewhere [31, 32].

#### Salt and vascular dysfunction

As mentioned previously, vascular dysfunction, including endothelial dysfunction with impaired vasodilatation, arterial stiffening, hypertrophy, and the proliferation of VSMCs, plays an important role in the etiology of hypertension. High-salt levels can contribute to these adverse mechanisms. In addition, there is circumstantial evidence that sodium can accumulate around endothelial cells [136]. Sodium enters endothelial cells through endothelial sodium channels (EnNaC), where it stabilizes actin filaments within the actin network beneath the plasma membrane, causing mechanical stiffening of the endothelial cell [137]. Additionally, high sodium concentrations increase the abundance of EnNaC in the endothelial cell membrane. EnNaC in turn is thought to further stabilize actin filaments through direct biomechanical interactions, as was shown for the ENaC alpha subunit [138]. Stiff endothelial cells are less deformable by blood stream alterations and subsequently release less NO. High intracellular sodium further impairs NO production by inhibiting endothelial NO synthase. Reduced NO release from endothelial cells enhances VSMC-mediated vasoconstriction and all arterial stiffening. High sodium concentrations also impact the endothelial glycocalyx (eGC). The eGC works as a shear stress sensor with subsequent inward cell signaling and as a sodium buffer, attracting the positively charged sodium ion to negatively charged proteoglycans [137]. In response to prolonged salt overload, the sodium buffer capacity of the eGC diminishes, and structural changes in heparan sulfate residues damage the eGC structure, impairing the endothelial sodium barrier and increasing sodium absorption into the cell [139]. Damage and conformational changes in the eGC further influence intracellular signaling and contribute to sodium-induced mechanical changes in endothelial cells. Chronic changes in mechanical properties and reduced NO release ultimately lead to the transition from endothelial function to dysfunction, increasing TGF-β1-mediated fibrogenesis, arterial stiffness and the development of hypertension [137, 140]. These salt-induced changes in the endothelium can be prevented by EnNaC inhibition, either directly by amiloride or indirectly by spironolactone [137].

#### Salt and (extra)renal RAAS

The sodium concentration in the distal tubulus is sensed by tubular epithelial cells of the macula densa via ion channels, particularly NKCC2 and potentially NHE2 [141]. Low sodium concentrations lead to the formation of prostanoids such as prostaglandin E_2_ (PGE_2_) and prostacyclin (PGI_2_), NO and adenosine triphosphate (ATP), which stimulate renin secretion by juxtaglomerular cells of the afferent arteriole and thereby initiate RAAS-mediated increases in BP [141]. The BP increase induced by RAAS activation is mediated by an increase in renal sodium reabsorption, among other pleiotropic effects. While high sodium intake suppresses renin secretion and the systemic RAAS under healthy conditions, a study with a mouse model of chronic kidney disease on a high-salt diet showed upregulation due to mechanisms other than systemic RAAS [141, 142]. On a high-salt diet, 5/6 nephrectomized mice exhibited activation of intrarenal RAAS stemming from renal tissue other than juxtaglomerular cells and increased expression of angiotensin-converting enzyme (ACE)-1, Ang II, and AT_1_ receptors. Additionally, neuronal expression of Ang II and AT_1_ receptors in brain regions that are important for cardiovascular regulation, including the organum vasculosum of the lamina terminalis, was enhanced [142]. Independent of preexisting kidney disease, sodium also affects neuronal activity within the organum vasculosum directly through sodium concentrations in cerebrospinal fluid; elevated sodium concentrations in the organum vasculosum of Sprague-Dawley rats increased sympathetic nerve activity and BP in a concentration-dependent manner [143].

#### Salt and oxidative stress

Oxidative stress and ROS influence the cardiovascular system, including the vasculature, heart, kidney, and nervous system, through pleiotropic effects, altering oxidative posttranslational modifications and redox signaling. ROS directly inactivate NO and subsequently inhibit vasodilatation. This disturbance in NO-dependent relaxation of VSMCs is further impaired by high sodium concentrations, which inhibit the activity of endothelial NO synthase. Additionally, high sodium intake enhances NAD(P)H oxidase activity and decreases superoxide dismutase expression in the renal cortex in rats, increasing local ROS generation [144]. Increased ROS can activate neuronal signaling in renal afferents and subsequently induce further peripheral vasoconstriction [9]. Accordingly, salt-sensitive rats show increased renal medullary oxidative stress due to excess superoxide generated by NOX, and NOX4 is especially important for salt-induced renal injury and hypertension [24, 145]. Recently, there has been emerging evidence that ROS contribute to immune activation in hypertension [24, 25].

#### Salt, the microbiome, and inflammation

By interacting with the external environment and internal metabolism, the gut microbiome plays an important role in priming the immune system. Furthermore, the gut microbiome is essential for the digestion of food, as well as the production of vitamins and other bioactive metabolites. Altogether, this leads to the suggestion that the gut microbiome is an endocrine organ and has crucial effects on other organs and complex systems within the body [146]. Understanding the individual components and bioactive metabolites of the gut microbiota in health and disease holds great potential for the development of new drug targets and (nutritive) interventions for several diseases. Studies have shown reduced microbial diversity in patients and mice with arterial hypertension, which is known as dysbiosis [146]. Partially controversial data even showed an association between colonization with certain microbial species and high BP [146]. Moreover, stool transplantation from hypertensive patients to germ-free mice led to a significant increase in BP in these mice compared to mice transplanted with fecal microbiotas from normotensive humans [147]. However, after comparing germ-free and conventionally raised mice, Karbach et al. found a reduction in the upregulation of RORγt, the signature transcription factor for IL-17 synthesis, and the attenuation of blood pressure increases in response to Ang II in germ-free mice [148]. This finding suggests that the gut microbiota facilitates Ang II-induced hypertension by IL-17-driven inflammation [148]. The link between the gut microbiome and hypertension was elegantly investigated by Wilck et al. who demonstrated the depletion of *Lactobacillus murinus* induced by high-salt intake in mice and in humans [149]. The reduction in *L. murinus* led to a reduced production of indoles. Indoles were shown to influence the differentiation of CD4^+^ cells into T_H_17 cells in a dose-dependent manner; more indoles were associated with the less polarization into T_H_17 cells. Accordingly, T_H_17 cells were expanded among lymphocytes in the intestinal lamina propria in mice that were fed a high-salt diet and had depleted *L. murinus* and reduced indole production, and this effect was absent in germ-free mice. Furthermore, treatment with *L. murinus* in mice fed a high-salt diet attenuated these changes and reduced salt-sensitive hypertension and autoimmune encephalomyelitis by modulating the T_H_17 cell population [149]. Expansion of T_H_17 cells due to a high-salt diet and a concurrent reduction in *L. murinus* could also affect other immune-mediated diseases. The exacerbation of experimentally induced colitis by high-salt intake was associated with a decrease in *L. murinus*, an increase in T_H_17 cells in the mesenteric lymph nodes and enhanced expression of proinflammatory genes related to IL-17 in immune cells of the lamina propria in the colon [150]. Furthermore, intestinal levels of the short-chain fatty acid butyrate were reduced; this bacterial metabolite is known to have an anti-inflammatory effect on intestinal macrophages, induce the differentiation of Treg cells in the colon and influence T-cell differentiation in vitro by inhibiting T_H_17 cell polarization [151–153]. Transferring butyrate-exposed T cells into mice led to the development of an attenuated form of colitis compared to the transfer of nonbutyrate-exposed T cells [151, 153]. Additionally, Faraco et al. showed IL-17-dependent endothelial and cognitive dysfunction in mice fed a high-salt diet [154]. Accordingly, the short-chain fatty acid propionate significantly attenuated systemic inflammation, cardiac hypertrophy, fibrosis, vascular dysfunction, and arterial hypertension in Ang II-infused wild-type and apolipoprotein E-knockout mice [155]. This effect was abolished in Ang II-infused mice that lacked Tregs, suggesting that Tregs mediate these anti-inflammatory effects [155].

An important and interesting observation was recently made by Rosshart et al. [156]. The mammalian phenotype is driven by the combination of the host genome and the microbial genome (microbiome), which is referred to as the metagenome. However, the repertoire of microbes in the wild has not been replicated in the laboratory. This difference can substantially distort the development and function of the immune system under laboratory conditions, leading to false translational assumptions of how our own immune system works. Thus, laboratory mice are too far from natural environmental conditions to faithfully mirror the physiology of humans. To address this shortcoming, embryos of laboratory mice can be transferred into wild mice to generate wildlings that resemble the natural mammalian metaorganism while preserving the research benefits of the tractable genetics of laboratory mice [146]. To our knowledge, hypertension has not yet been examined in wildlings. More work needs to be done to clarify the role of gut bacteria in hypertension.

### Impact of salt: outlook

The importance of daily salt intake was underscored recently by the results of the Salt Substitute and Stroke Study in China, which examined the effect of substituting table salt, and the researchers examined 100% sodium chloride and substitutes containing 75% sodium chloride and 25% potassium chloride over a period of 5 years [157]. The authors found a reduction in systolic BP by approximately 3.3 mmHg and decreased risks of stroke, severe cardiovascular events, and overall mortality by ~14%, 13% and 12%, respectively [157]. High potassium intake may lower blood pressure and reduce salt sensitivity. Accordingly, a prospective cohort study assessed sodium and potassium intake that was measured in 24 h urine samples and showed a dose-dependent association between higher sodium and lower potassium intakes with an increased cardiovascular risk [158]. To explain this finding, a potassium switch in the distal nephron was proposed recently that permits homeostasis despite variations in sodium intake [113]. A new aspect of high sodium intake and its impact on water homeostasis has been discussed recently [159]. Increased renal salt excretion and water conservation in response to high dietary salt intake results in katabolic metabolism. Sodium excretion in parallel with water reabsorption requires extrarenal urea production in the liver and skeletal muscles, as well as urea recycling in the kidneys; both processes require high energy consumption [160]. To provide for the increased tubular energy consumption, endogenous protein sources such as skeletal muscle are exploited or increased food intake is needed [161]. Overall, salt plays a pivotal role in the etiology of arterial hypertension and cardiovascular disease.

## Aldosterone, mineralocorticoid receptor, inflammatory cells, and hypertension

**Fig. 4:**
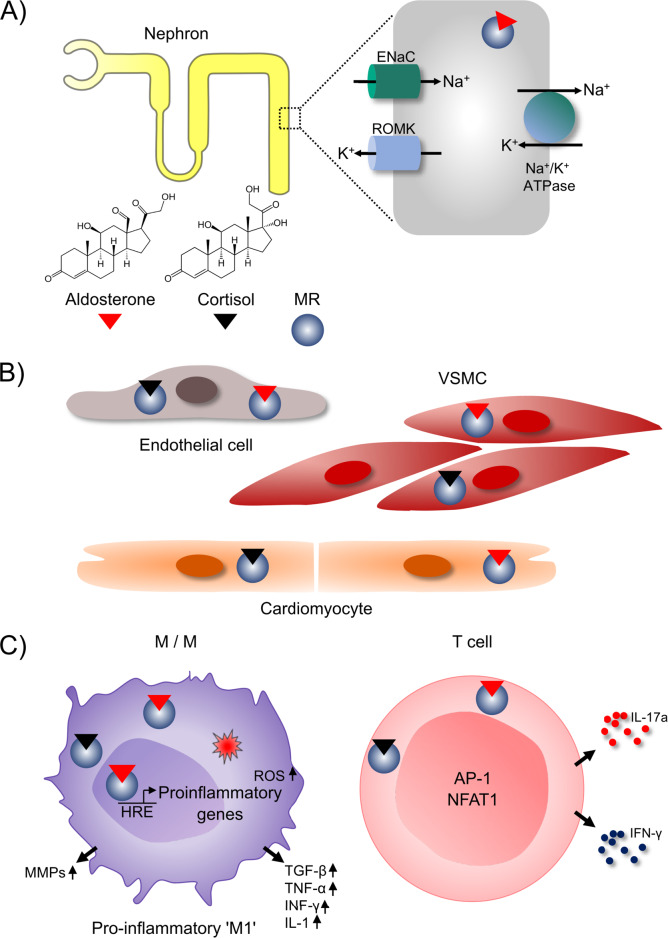
**A** The classic effect of the MR is to promote renal sodium reabsorption by increasing the abundance of the epithelial sodium channel (ENaC) on the apical membrane of epithelial cells in the collecting duct after binding to its classic ligand aldosterone. **B** Second, it became clear that MR signaling in endothelial cells, VSMCs and cardiomyocytes promotes hypertension and fibrosis. **C** Third and more recently, it was discovered that MR signaling in innate and adaptive immune cells can cause hypertension and hypertensive end-organ damage. The effect of mineralocorticoid is conveyed via the ligand-activated translocation of cytosolic MR into the nucleus, where it binds to a specific DNA sequence known as the hormone response element (HRE) and initiates proinflammatory gene expression, inducing an M1 cascade with increased generation of ROS and M1 markers, as well as the secretion of MMPs and cytokines. In T cells, MR interacts with NFAT1 and AP-1 and promotes differentiation toward IFN-γ- and IL-17A-producing T-cell populations. ENaC epithelial sodium channel, ROMK renal outer medullary potassium channel, MR mineralocorticoid receptor, VSMC vascular smooth muscle cell, M/Ms monocytes/macrophages, MMP matrix metalloprotease, HRE hormone response element, ROS reactive oxygen species, TGF transforming growth factor, TNF tumor necrosis factor, INF interferon, IL interleukin, AP-1 activator protein 1, NFAT1 nuclear factor of activated T cells 1

The history of aldosterone and the mineralocorticoid receptor (MR) can be divided into three phases. First, aldosterone and the MR are mainly known for their classic effects: promoting renal sodium and water reabsorption by inducing epithelial sodium channels (ENaC) in epithelial cells in the distal nephron in response to aldosterone binding, thereby regulating BP and salt homeostasis, as shown in Fig. 4A [162]. However, in the last decade, it has been shown that MR is present on virtually all cells of the cardiovascular system, such as endothelial cells, VSMCs and cardiomyocytes [162, 163] (Fig. 4B). Experimental studies using cell type-specific gene targeting of the MR in mice have revealed the importance of this extrarenal aldosterone signaling in cardiomyocytes, endothelial cells and VSMCs in hypertension and CVD. MR activation in vascular smooth muscle cells (Fig. 4B) directly contributes to vascular oxidative stress, vasoconstriction, and arterial hypertension, as McCurley et al. showed by selectively knocking out MR in vascular smooth muscle cells in mice [164]. Finally, in the third phase, it became clear that MR also plays an important and proinflammatory role in the innate and adaptive immune systems. The effect of mineralocorticoid is conveyed via the ligand-activated translocation of cytosolic MR into the nucleus, where it binds to a specific DNA sequence known as the hormone response element and initiates proinflammatory gene expression (Fig. 4C).

### The MR in innate immune cells

The presence of the MR in monocytes/macrophages (M/Ms) has been shown at the mRNA and protein levels [165–167]. Aldosterone infusion in rodents promotes renal macrophage infiltration and macrophage differentiation toward the proinflammatory M1-like phenotype and enhances the expression of markers associated with M1-like activation, such as TNF-α and inducible nitric oxide synthase (iNOS) [41, 162, 168]. This aldosterone-induced M1-like activation is reduced by aldosterone antagonists [168]. MR signaling in M/Ms increases the expression of markers linked to M1-like activation, ROS formation and the secretion of inflammatory cytokines and matrix metalloproteases (MMPs) (Fig. 4C) [165, 169]. In contrast, myeloid-specific knockout of MR or MR antagonist treatment skews cells toward an M2-like anti-inflammatory phenotype and induces specific M2-like markers such as IL-10, Arg1 and peroxisome proliferator-activated receptor (PPAR-γ). The role of the myeloid MR has been studied in different models of hypertension. Knockout of MR in monocytes and macrophages uniformly decreased vascular and cardiac fibrosis [165, 170], independent of the model of hypertension used. Cardiac hypertrophy and fibrosis induced by aortic constriction were also significantly attenuated in myeloid MR-deficient mice. Macrophage infiltration in the heart was inhibited, and the expression of inflammatory genes was decreased in myeloid MR-deficient mice. In addition, aortic fibrosis and inflammation were attenuated [171]. MR-knockout mice showed a smaller increase in BP and cardiac fibrosis than wild-type mice after 8 weeks of DOCA salt treatment. In summary, these studies clearly demonstrate a role for MR in macrophages in hypertension and hypertensive cardiovascular remodeling [67].

### The MR in adaptive immune cells

**Fig. 5:**
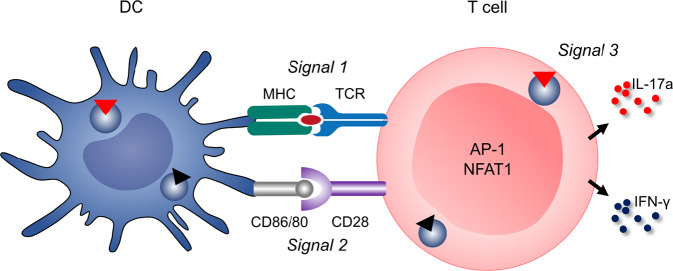
MR activation as a third signal in the immunological synapse in T-cell activation. T cells require three signals for activation: signals 1, 2, and 3. Signal 1 is T-cell receptor recognition of specific antigenic peptides presented on APCs. Signal 2 is costimulation, which involves interactions between CD80 and CD86 on APCs and CD28 on T cells. Signal 3 involves the stimulation of receptors outside of the immunologic synapse by hormones, cytokines, and danger signals present in the inflammatory milieu. MR signaling is a new signal 3 for lymphocytes associated with arterial hypertension. MR on T cells stimulates the production of IFN-γ, resulting in hypertension and cardiovascular fibrosis. DC dendritic cell, MHC major histocompatibility complex, TCR T-cell receptor, CD cluster of differentiation, AP-1 activator protein 1, NFAT1 nuclear factor of activated T cells 1, IL interleukin, IFN interferon

MR expression in lymphocytes has been shown by Armanini et al. by radioreceptor assays [172]. Moreover, the expression of MR in T cells has been demonstrated by FACS analysis, PCR and Western blotting [173]. Therefore, convincing data exist that MR is expressed in T cells. Li et al. and Sun et al. found an important role for MR in T cells in two studies. First, the researchers showed that T-cell-specific MR knockout in mice resulted reduced cardiac hypertrophy, fibrosis and dysfunction compared to those in control mice after abdominal aortic constriction [171]. Correspondingly, the mice exhibited reduced cardiac inflammation, which was illustrated by decreased accumulation of myeloid cells and reduced expression of inflammatory cytokines. Flow cytometry showed that T-cell-specific MR-knockout mitigated the Ang II-induced accumulation of IFN-γ-producing T cells, particularly the CD8^+^ population, in both the kidneys and aorta. At the molecular level, MR interacted with NFAT1 and activator protein-1 (AP-1) in T cells (Fig. 4C) [173]. Barbaro, Kirabo and Harrison proposed in response to these data that MR signaling  is a so called signal 3 in T cells.  [174]. T cells require three signals for activation, called signals 1, 2, and 3 (Fig. 5). T-cell receptor recognition of specific antigenic peptides presented on APCs constitutes signal 1. Signal 2 is costimulation, which involves interactions between CD80 and CD86 on APCs and CD28 on T cells. Signal 3 involves the stimulation of receptors outside of the immunologic synapse by hormones, cytokines, and danger signals present in the inflammatory milieu [174]. Therefore, MR signaling is clearly a new signal 3 for lymphocytes in arterial hypertension [67].

### Type 1 angiotensin receptor in immune cells

**Fig. 6:**
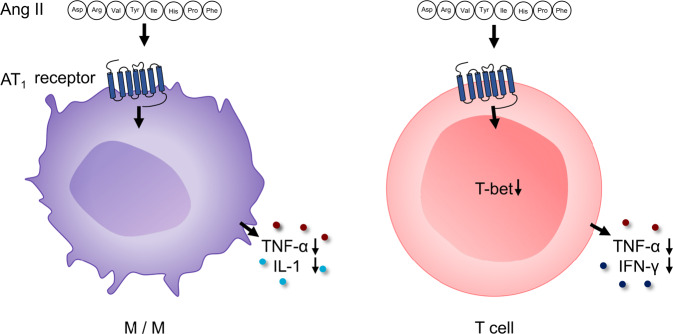
Anti-inflammatory effect of AT_1_ receptor activation on inflammatory cells. Ang II angiotensin II, AT_1_ receptor angiotensin type 1 receptor, TNF tumor necrosis factor, IL interleukin, IFN interferon, M/Ms monocytes/macrophages

The most important part of the RAAS is undoubtedly the AT_1_ receptor. Although classically known for its role in regulating circulatory homeostasis, Ang II acts as a powerful proinflammatory mediator by stimulating the AT_1_ receptor. The binding of Ang II to its receptor results in nuclear factor (NF)-κB and ROS activation. This activation increases vascular permeability and upregulates adhesion molecules, chemokines and inflammatory cytokines such as TNF-α and IL-6 [175]. The AT_1_ receptor is expressed on almost all cells in the body. However, very little data are available on the role of the AT_1_ receptor in inflammatory cells in hypertension. The AT_1_ receptor is expressed on myeloid cells and lymphocytes. Surprisingly, activation of the AT_1_ receptor in these cells has immunologic effects that diverge from AT_1_ receptor stimulation in the kidney, heart, or vasculature. AT_1_ receptors on immune cells appear to play a protective role, as reviewed by Crowley and Rudemiller and summarized in Fig. 6 [23].

In an Ang II infusion model of hypertension, AT_1_ receptor–deficient bone marrow chimeras have exaggerated BP elevation, albuminuria, and accumulation of T cells and macrophages in the kidney [176]. In the same Ang II infusion model, mice lacking myeloid AT_1_ receptors have a preserved hypertensive response but more severe renal tubular injury and interstitial fibrosis than controls [177]. The capacity of macrophage AT_1_ receptors to mitigate kidney fibrosis was similarly evident in a normotensive model of kidney fibrosis known as the ureteral obstruction model [177]. Activating the AT_1_ receptor on macrophages suppresses their proinflammatory M1 polarization and reduces TNF-α and IL-1β expression.

Ang II-induced hypertension in T-cell-specific AT_1_ receptor-knockout mice resulted in similar BP responses but augmented albuminuria and exaggerated perivascular accumulation of CD4^+^ T lymphocytes in the hypertensive kidney, as reviewed in [23]. CD4^+^ T cells lacking the AT_1_ receptor were isolated from the hypertensive kidney or spleen and expressed increased levels of the proinflammatory cytokines IFN-γ and TNF-α and the T_H_1 transcription factor T-bet (Tbx21), which drives the expression of these cytokines in T cells [178]. Thus, AT_1_ receptor stimulation in T cells suppresses T-bet-dependent differentiation of CD4^+^ T-helper cells toward the proinflammatory T_H_1 cell lineage (Fig. 6).

Thus, based on bone marrow chimera and conditional gene targeting studies, AT_1_ receptors on myeloid and lymphoid populations play an immunomodulatory role that tempers the pathogenic actions of AT_1_ receptors in the kidney and vasculature during hypertension [23]. Interestingly, immune amplification by AT_1_ receptor blockade resulted in a severe, autoimmune-mediated enteropathy associated with olmesartan treatment in rare cases [179, 180]. The reason for the opposite effects of MR and AT_1_ receptor activation in inflammatory cells remains unclear. One possible advantage of this counterintuitive finding was postulated by Crowley and Rudemiller [23]: the protective effect of AT_1_ receptors on immune cells may provide a feedback mechanism to alleviate or limit the proinflammatory effects of AT_1_ receptor activation in target organs. Once immune cells are recruited into tissue that is damaged by AT_1_ receptor activation, these cells can dampen inflammation and limit the severity of injury.

## Conclusion

Approximately 80 years have passed since Page proposed the mosaic theory of arterial hypertension. Since then, several new, exciting, and sometimes surprising findings and connections have been added. These findings prompted us to propose a revised version of the mosaic theory that is shown in Fig. 1. Although immune cells and high-salt intake are important for protecting against invading pathogens, their interaction and consequent overactivation may lead to tissue damage and high BP. Clearly, salt and inflammation play a central role in almost every aspect of hypertension and therefore deserve a place in the center of the mosaic. In humans, verification of the experimental evidence suggesting a relationship between salt intake, immune cells, inflammation and hypertension is a major challenge, but an improved understanding of how salt and immune cells influence hypertension is crucial and clinically relevant for identifying therapeutic interventions. Research in wildling mice, which have diverse microbial compositions compared to clean laboratory mice, may mimic human hypertension better than current experimental models and may have more translational relevance. A better and deeper understanding of the immune mechanisms of hypertension will allow for more targeted and personalized therapeutic approaches. The aim of investigating immunity in hypertension is to develop therapies that can be tested in vivo. Novel technologies, such as targeted nanoparticles, cell-specific siRNA or nanobodies, may be able to inhibit or overexpress certain factors in specific subsets of immune cells, thus delivering precise and targeted therapies without the risks of global immunosuppression [76]. In this context, MR (but not the AT_1_ receptor) in myeloid or lymphoid cells could be a target of interest. There are remaining knowledge gaps to be addressed before immunomodulatory therapies might be applied to even a subset of patients with hypertension. In contrast, reducing salt intake or substituting sodium with potassium are easy to achieve and successful in reducing BP and hypertensive end-organ damage [157]. This reduction in BP and morbidity is predicted by the revised mosaic theory that places salt and inflammation in the center of hypertension.
